# Serological cytokine profiles of cardiac rejection and lung infection after heart transplantation in rats

**DOI:** 10.1186/s13019-019-0839-5

**Published:** 2019-01-29

**Authors:** Hao Chen, Juhua Yang, Shengchao Zhang, Xuan Qin, Wei Jin, Lihua Sun, Feng Li, Yunfeng Cheng

**Affiliations:** 1 Department of Thoracic Surgery, Zhongshan Hospital Qingpu Branch, Fudan University, Shanghai, China; 2 Department of Hematology, Zhongshan Hospital Qingpu Branch, Fudan University, 1158 East Parkway, Shanghai, 201700 China; 3 Department of Hematology, Zhongshan Hospital, Fudan University, Shanghai, China; 4 Institute of Clinical Science, Zhongshan Hospital, Fudan University, 180 Fenglin Rd, Shanghai, 200032 China; 5 Shanghai Institute of Clinical Bioinformatics, Fudan University Center for Clinical Bioinformatics, Shanghai, 200032 China

**Keywords:** Heart transplant, Acute rejection, Pulmonary infection, Cytokine profile, Differential diagnosis

## Abstract

**Background:**

Allograft rejection and infection are the major sources of morbidity and mortality after heart transplant. Early differential diagnosis is clinically crucial but difficult. The aim of the study was to examine serum cytokine profiles associated with each entity and whether such profiles could help to differentiate between them.

**Methods:**

Heart allografts from Wistar rats were transplanted to Lewis rats as described by Yokoyama. Cardiac rejection and pulmonary bacterial infection were induced by Cyclosporine cessation and bacteria bronchus injection, and pathologically confirmed. Ninety serological cytokines profiles of the study objects were then simultaneously measured using a biotin label-based cytokine array. The fold change (FC) was used for relative cytokine concentration comparison analysis.

**Results:**

Four cytokines in cardiac rejection group were significantly dysregulated as compared to health controls (β -Catenin, 0.51 FC; E-Selectin, 0.62 FC; IFN-gamma, 1.87 FC; and IL-13, 0.60 FC, respectively). In pulmonary infection animals, 11 cytokines were remarkably dysregulated in comparison with the control group (CINC-3, 0.57 FC; CNTF R alpha, 0.59 FC; E-Selectin, 0.58 FC; FSL1,0.62 FC; Hepassocin, 0.64 FC; IL-2, 0.26 FC; IL-13, 0.49 FC; NGFR, 0.57 FC; RAGE, 0.50 FC; TIMP-1, 0.49 FC; and IFN-gamma, 1.77 FC, respectively). Eleven cytokines were significantly up-regulated in cardiac rejection group comparing to the pulmonary infection animals (FSL1, 2.32FC; Fractalkine, 1.65FC; GFR alpha-1, 1.64FC; IL-2, 2.72FC; IL-5, 1.60FC; MMP-2, 1.71FC; NGFR, 2.25FC; TGF-beta1, 1.58FC; TGF-beta3, 1.58FC; Thrombospondin, 1.64FC, and TIMP-1, 1.52FC, respectively).

**Conclusions:**

The current study illustrated the disease-specific serological cytokine profiles of allograft rejection and pulmonary bacterial infection after cardiac transplant. Such disease associated cytokine portraits might have the potential for early discrimination diagnosis.

## Background

Heart transplantation remains the only established cure for patients suffer from end-stage cardiac diseases. Acute cardiac allograft rejection and infection are the major causes of death for cardiac transplanted patients, accounting for almost 40% reported deaths within the first year after the surgery [1]. The clinical therapy of rejection and infection is closely intertwined, immunosuppression to prevent or treat rejection can increase the risk of infection; tapering off immunosuppressant to treat infection can exacerbate rejection. As both entities present with similar, non-specific symptoms such as dysponea, fatigue, and low-grade fever at disease onset, their early discrimination is critical yet challenging.

Expression profiling has the potential not only to identify disease biomarkers, but also may provide mechanism insights. Studies have utilized either genomics or proteomics to examine heart rejection [2, 3]. However little has been done to investigate the complex pathophysiology and underlying mechanisms of cardiac rejection and infection that complicating heart transplant. Using an animal model of lung bacterial infection after heterotopic cardiac transplantation we have recently developed [4], the current study simultaneously examined ninety serological cytokines profiles in the course of acute cardiac rejection and pulmonary infection with a biotin-labeled-based cytokine protein array system. Our hypotheses are 1) disease-specific cytokine pattern exists in each entity, and 2) such cytokine patterns might have the potential to aid differential diagnosis.

## Methods

### Animal care

Inbred Wistar (donor) and Lewis (recipient) rats, male, weighing 200–250 g, were purchased from Slac Laboratory (Shanghai, China), maintained in the animal facility of Zhongshan hospital, Fudan University under standard conditions, and allowed to drink water and eat rodent chow ad libitum. The current study was approved by the Animal Care and Use Committee (ACUC) of Zhongshan hospital, Fudan University. Animal care followed the criteria of the ACUC of the NIH. Throughout the study, every effort was taken to minimize any suffering of the animals.

### Heterotopic cardiac transplantation

Cardiac allograft rejection and pulmonary bacterial infection were induced as the following [4]. Cardiac transplantations were performed using a modified version of the heterotopic cardiac transplantation model reported by Yokoyama et al [5]. In brief, animals were anesthetized with 40 mg/kg of intraperitoneal injection of pentobarbital. After heparinization, donor heart was procured in the conventional fashion. After flushing the coronary circulation with 10 ml of cold lactated Ringers solution, donor heart was placed in iced lactated Ringers solution where the donor heart was tailored by the ligation of main pulmonary artery, superior vena and inferior vena cave (IVC), and the removal of tricuspid valve and interatrial septum. After such preparations, donor hearts were replaced in iced lactated Ringers solution while the recipient animals were prepared. The IVC and abdominal aorta of the recipient animals are exposed through a longitudinal laparotomy with a left subcostal take-off. The donor ascending aorta was then anastamosed to the recipient abdominal aorta with a running 8–0 prolene. Similarly, the donor right atrium was anastamosed to the recipient IVC. Consequently, the left atrium and ventricle of graft were loaded with the blood from the right atrium through the interatrial communication. Upon re-establishment of blood flow, transplanted heart resumed spontaneous contractions in sinus rhythm, and was free of gross surgical injury at the time of closure.

### Induction of pulmonary infection

Via a tracheostomy, 0.2 ml of *Pseudomonas aeruginosa* ATCC 27853 (1 × 10^9^ CFUs/ml, American Type Culture Collection, Manassas, VA) was injected into the stem bronchus of recipient animals assigned to the infection group under direct vision to induce bacterial pneumonia. For non-infection animals, 0.2 ml of normal saline was injected into the stem bronchus of recipient rats under direct vision.

### Animal grouping and sample procurement

**Fig. 1:**
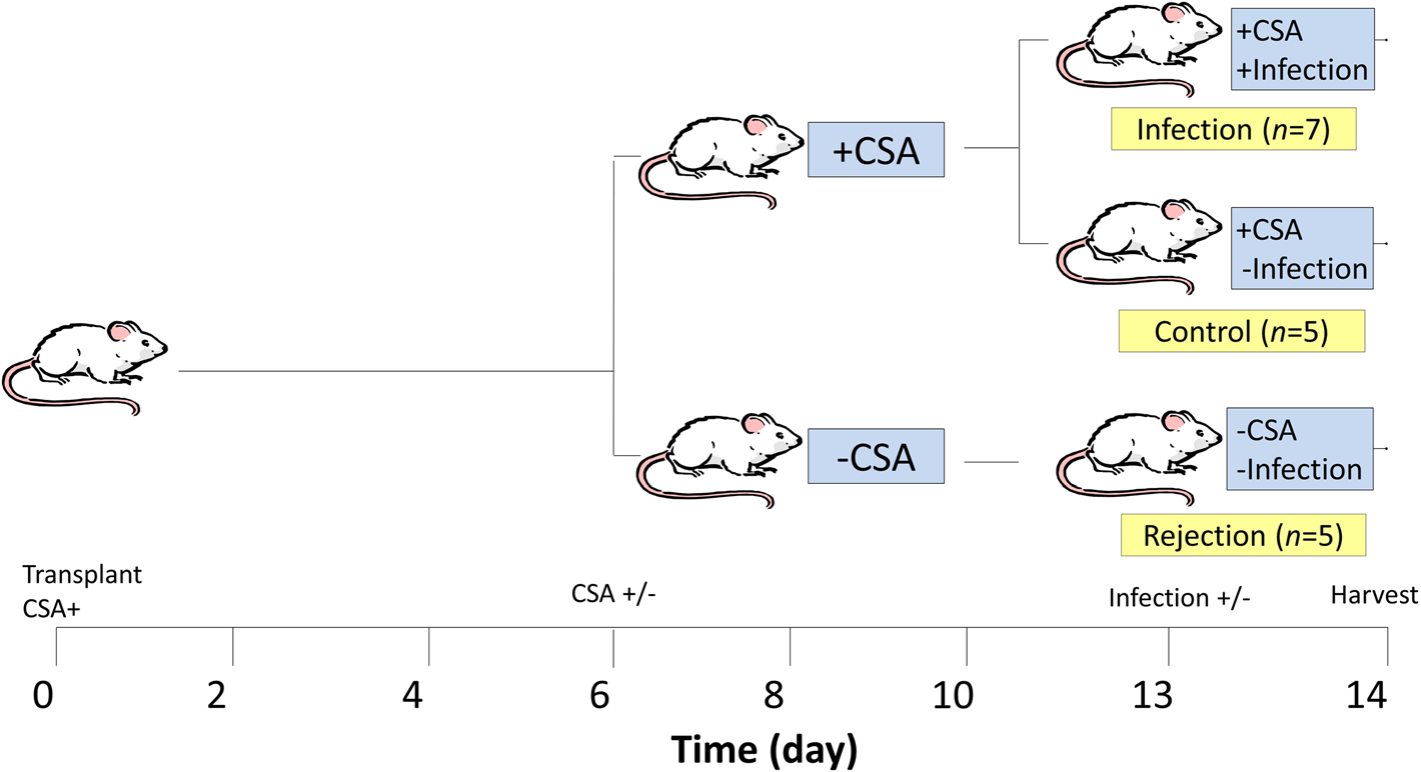
Study design and animal grouping. All recipient animals received daily cyclosporine A (CSA) subcutaneous injection to suppress rejection on post-operative day (POD) 0. On POD 6, recipient animals were randomized to either have their CSA continued, or have their CSA discontinued and began on a normal saline placebo injection (rejection group, *n* = 5). On POD 13, non-rejection animals were further randomized to either receiving intratracheal inoculation of Pseudomonas aeruginosa (infection group, *n* = 7), or receiving intratracheal inoculation of normal saline (control group, n = 5)

All recipient animals were begun on daily cyclosporine A (CSA) subcutaneous injection (10 mg/kg/day) to suppress rejection. On post-operative day (POD) 6, recipient animals were randomized to either have their CSA continued, or have their CSA discontinued and began on a normal saline placebo injection (10 mg/kg/day subcutaneously, rejection group, *n* = 7). On POD 13, non-rejection animals were further randomized to either receiving intratracheal inoculation of *Pseudomonas aeruginosa* (infection group, *n* = 7), or receiving intratracheal inoculation of normal saline (control group, *n* = 7). Animals of the rejection group also received intratracheal inoculation of normal saline on POD 13 (Fig. 1).

Graft viability was assessed daily by palpation of the donor heart. Rejection was defined as cessation of a palpable heartbeat and was confirmed by direct inspection at laparotomy upon organ harvest. Animals were sacrificed on POD 14, lungs and transplanted hearts were procured after blood withdrawals. Cross-sections of heart and lung were processed for histopathology using hematoxylin and eosin staining. Histological changes were blindly assessed by a pathologist, allograft rejections were evaluated using the International Society of Heart and Lung Transplantation (ISHLT) system for rejection [6].

### Measurement of cytokines

Upon harvest on POD 14, peripheral blood samples were withdrawal from all recipient animals. After being allowed to clot at room temperature for 1 h, blood samples were centrifuged at 1500×g for 10 min, sera were then collected and stored at − 80 °C until use.

Serum levels of 90 cytokines were measured by RayBio Biotin Label-based Rat Antibody Array 1 (RayBiotech, Norcross, GA, USA) follow the recommended protocol from manufacture. In brief, sample mixtures consist of serum aliquots from the same study groups were biotinylated and dialyzed for incubation with the array. These samples were then added to the array membrane and incubated at room temperature. After incubation with HRP-stretavidin, the signals were visualized by exposure to x-ray film with subsequent development. Cytokines of interest were quantified by densitometry using ImageJ software (http://imagej.net). By subtracting the background staining immediately surrounding each sample and normalizing to the positive controls on the same membrane, the relative protein concentrations were obtained. In precaution of inter-assay variations, cytokines levels were measured using cytokine array kit from the same shipment, and performed under the same laboratory conditions.

### Statistical analysis

Samples with cytokine levels below the detection limits were arbitrarily assigned the values corresponding to the minimum limits. Per manufacture’s instruction (https://www.raybiotech.com/files/manual/Antibody-Array/AAR-BLG-1.pdf), the relative protein concentration of cytokine was used for comparison analysis. Each cytokine was compared between groups in fold change (FC). A FC of 1.50 or greater represents significant up-regulation, and FC of 0.65 or less represents significant down-regulation.

## Results

### Induction of allograft rejection and pulmonary infection

The mean total transplant operation time was 55 min (range, 48–65 min). The mean ischemic time of donor hearts was 35 min (range, 31–47 min). The allograft heartbeat of the non-rejection animals remained energetic throughout the study period, while the transplanted heart gradually lost its contractions over time after CSA cessation in the rejection group. Major morbidities of myocardial infarction (*n* = 1, rejection group; *n* = 2, control group, respectively) and thrombosis (*n* = 1, rejection group) were detected by direct observation and histopathology, and these 4 animals were excluded from the study. The remaining 17 animals (*n* = 5, rejection group; *n* = 7, infection group; and *n* = 5, control group, respectively) were processed for further analysis.

Histological evaluation of cardiac allografts of the non-rejection group revealed minimal histologic changes with no evidence of interstitial edema or inflammation (ISHLT grade 0 R). In rejection group, however, severe rejection (ISHLT grade 3 R) were confirmed with diffuse inflammatory process and necrosis normal staining patterns observed on the tissue sections.

Similarly, minimal histological changes were found in the non-infection animal lung sections, while animals of the infection group had severe pneumonia with typical changes such as alveolar septal congestion, edema, focal alveolar hemorrhage, and infiltration of neutrophils and macrophages.

### Cytokine portraits in cardiac rejection animals

**Fig. 2:**
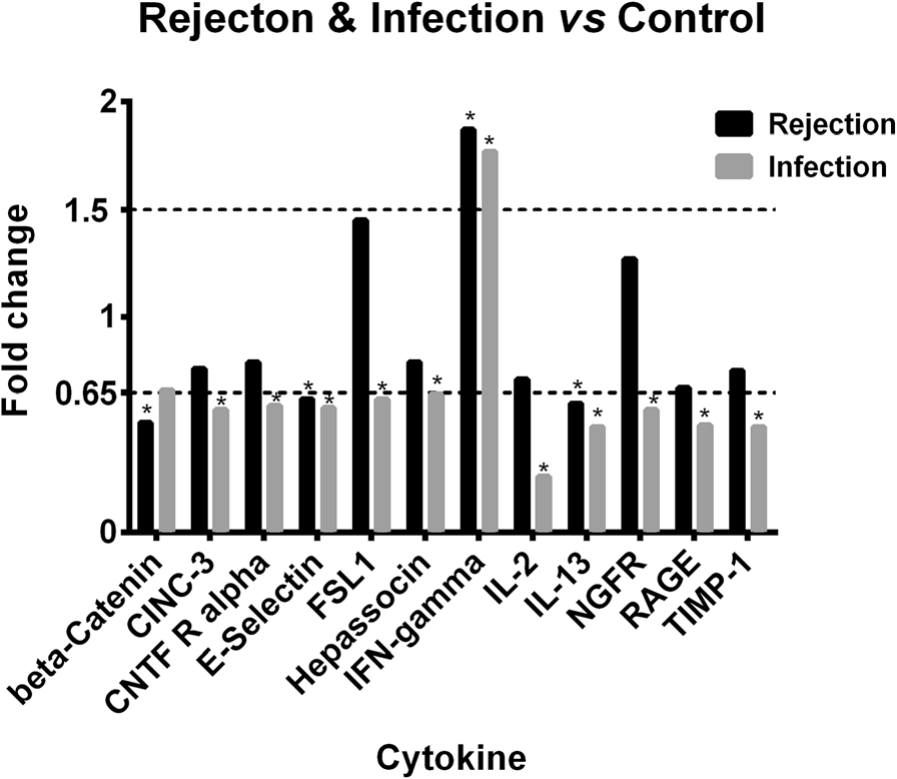
Serum level fold change of beta-Catenin, CINC-3, CNTF R alpha, E-Selectin, FSL-1, Hepassocin, IFN-gamma, IL-2, IL-13, NFFR, RAGE, and TIMP-1 in the groups of rejection and infection in comparison with those in the group of health control. The asterisk represents significant difference

Serum levels of 90 cytokines were analyzed simultaneously. The statistic significant differences were reached in 4 cytokines in rejection group as compared to the control group (beta-Catenin, 0.51 FC; E-Selectin, 0.62 FC; IFN-gamma, 1.87 FC; and IL-13, 0.60 FC, respectively) (Fig. 2). Of them, beta-Catenin, E-Selectin, and IL-13 were significantly down-regulated in the rejection group whereas serum level of IFN-gamma was remarkably up-regulated in the rejection group.

### Cytokine portraits in pulmonary infection animals

The statistic significant differences were reached in 11 cytokines when infection animals comparing to the health controls. Of them, 10 cytokines (CINC-3, CNTF R alpha, E-Selectin, FSL1, Hepassocin, IL-2, IL-13, NGFR, RAGE, and TIMP-1) were significantly down-regulated (CINC-3, 0.57 FC; CNTF R alpha, 0.59 FC; E-Selectin, 0.58 FC; FSL1,0.62 FC; Hepassocin, 0.64 FC; IL-2, 0.26 FC; IL-13, 0.49 FC; NGFR, 0.57 FC; RAGE, 0.50 FC; and TIMP-1, 0.49 FC, respectively), while IFN-gamma were up-regulated in the rejection group (1.77 FC) (Fig. 2).

### Differential cytokine patterns between cardiac rejection animals and pulmonary infection animals

**Fig. 3:**
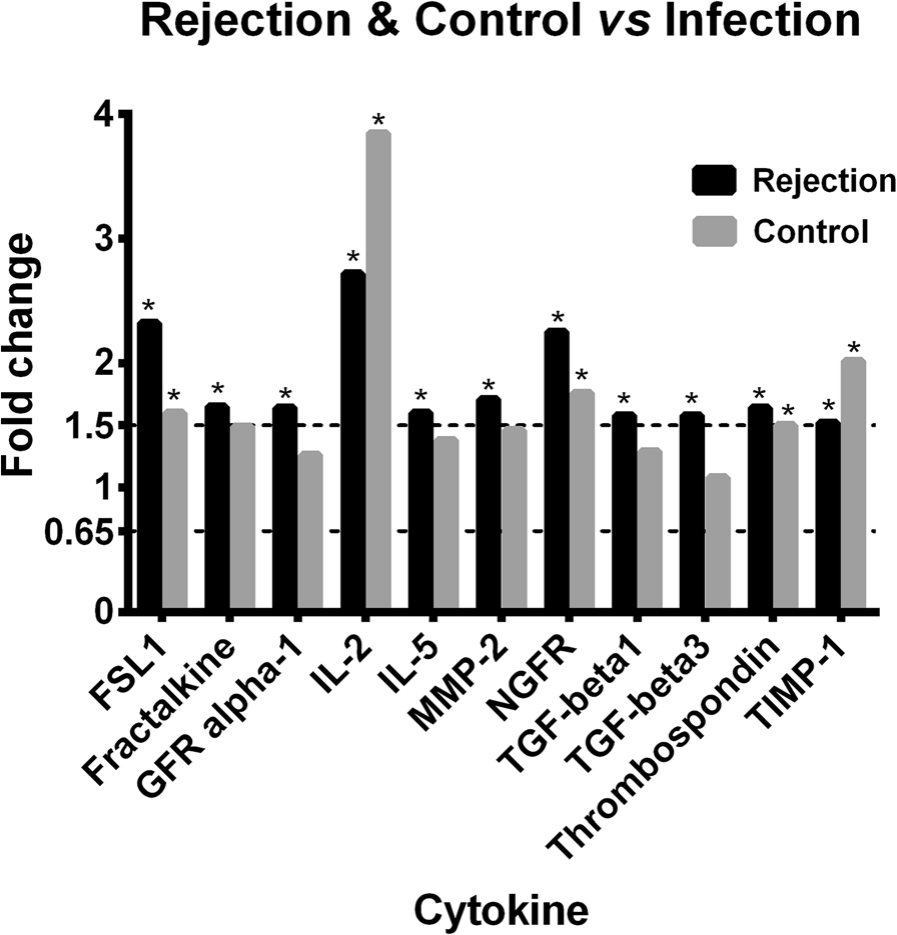
Serum level fold change of FSL1, Fractalkine, GFR alpha-1, IL-2, IL-5, MMP-2, NGFR, TGF-beta1, TGF-beta3, Thrombospondin, and TIMP-1in the groups of rejection and health control in comparison with those in the group of infection. The asterisk represents significant difference

Eleven cytokines (FSL1, Fractalkine, GFR alpha-1, IL-2, IL-5, MMP-2, NGFR, TGF-beta1, TGF-beta3, Thrombospondin, and TIMP-1) were found significantly up-regulated in cardiac rejection animals comparing to the pulmonary infection ones (Fig. 3). Of them, IL-2 exhibited the highest up-regulation with a fold increase of 2.72, followed by FSL1 (2.32 FC), and NGFR (2.25 FC). While the levels of IFN-gamma, IL-13 and E-Selectin were statistically dysregulated in both groups of rejection (1.87 FC, 0.60FC, and 0.62 respectively) and infection (1.77FC, 0.49FC, and 0.58, respectively) when compared to the health controls, no difference was found between the groups of rejection and infection, although numerically higher in the rejection group (1.06 FC, 1.24FC, and 1.08FC, respectively, in comparison with infection group) (Fig. 2).

## Discussion

Allograft rejection and infection are the leading causes of death after heart transplantation, ranks 4th and 1st respectively during the first year after the surgery, according to the latest ISHLT adult heart transplantation annual report [1]. Although treatment targeting rejection and infection have being continuously explored, the clinical outcomes remain unsatisfying. As both entities present with similar, nonspecific symptoms at early stage, it is difficult for timely discrimination, which lead to misdiagnosis and therapy delay. Endomyocardial biopsy as the gold standard for cardiac rejection, is invasive and biased. Although transcript expression profiling of peripheral blood has been reported for cardiac rejection surveillance, however little has been done to explore the potential role of serologic cytokine profiling to discriminate between infection and allograft rejection.

The assessment of 90 cytokines revealed 4 proteins (beta-Catenin, E-Selectin, IFN-gamma, and IL-13) in the serum of cardiac allograft rejection animals, and 11 proteins (CINC-3, CNTF R alpha, E-Selectin, FSL1, Hepassocin, IL-2, IL-13, NGFR, RAGE, IFN-gamma, and TIMP-1) in the serum of bacterial pulmonary infection animals that were remarkably dysregulated in comparison with the health controls. These results suggest that distinctive serum protein portraits do exist in allograft rejection and infection in the context of cardiac transplantation. Furthermore, levels of FSL1, Fractalkine, GFR alpha-1, IL-2, IL-5, MMP-2, NGFR, TGF-beta1 and -beta3, Thrombospondin, and TIMP-1 were significantly up-regulated in cardiac rejection animals in comparison with the pulmonary infection animals. However, none of them could differentiate rejection animals from health controls. These findings support our hypothesis that serological cytokine profiling might have the potential to differentiate between infection and rejection.

In the panel of cytokines that differentiates between infection and rejection, IL-2 exhibited the highest fold increase. Originally identified as a T cell growth factor, IL-2 is known to play a dual role in induction or suppression of immune responses via activation of conventional and regulatory T cells [7]. Researchers have found that IL-2 is increased in heart transplant patients and is specifically involved in cardiac rejection [8, 9]. During cardiac rejection, IL-2 mRNA and protein are predominantly produced by graft-infiltrating lymphocytes [10]. Its intracellular expression and soluble production has the potential to be served as a predictive biomarker for acute rejection and personal drug response [11].

FSL1 and NGFR succeed IL-2 as the top 3 remarkably increased cytokines rejection vs infection. FSL1 is a secreted glycoprotein produced expressed in various tissues. The essential biological function of FSL1 is binding and neutralizing transforming growth factor beta (TGF-beta) superfamily, enhancing synthesis of proinflammatory cytokines and chemokines by immune cells. FSL1 is elevated in various inflammatory conditions and decreased during the course of treatment, it may therefore be a valuable biomarker for such diseases [12, 13]. Its dysregulated expression postulate its potential role in contributing to allograft dysfunction following lung transplantation [14, 15]. The activity of the heart is widely regulated by its autonomous nervous system, however this important mechanism of control is lost in the transplanted heart. NGFR is reported to play key role in restoring innervation of the cardiac allograft, as such its upregulation might be a response to the impairment of the allograft nervous system caused by immune-rejection [16].

Serum levels of Fractalkine, GFR alpha-1, IL-5, MMP-2, TGF-beta1, TGF-beta3, Thrombospondin, and TIMP-1 were also up-regulated in rejection animals than those in infection animals. Among them, Fractalkine belongs to the chemokine family whose expression was found negligible in nonrejecting cardiac isografts but was significantly enhanced in rejecting allografts, its blockage significantly prolonged allograft survival, suggesting a critical role for Fractalkine in in the pathogenesis of acute rejection [17–19]. GFR alpha-1 is a coreceptor that recognizes the GDNF family of ligands and has been implicated in the regulation of neuronal cell survival and differentiation. It has a role in the progression and metastasis of human cancers in that it promotes migration and invasion [20, 21], however its role in cardiac rejection remains unclear. IL-5 plays an essential role in inflammation and the allergic response, favoring the production, maturation, proliferation, recruitment, differentiation, and survival of eosinophils. Over-expression of IL-5 significantly increases eosinophil numbers and antibody levels, its critical role has long been recognized in allograft rejection [22, 23]. IL-5 blockade with anti-IL-5 antibody could prevent chronic rejection of cardiac grafts [24]. MMP-2, a type IV collagenase, which facilitates tissue invasion of leukocytes and exerts a direct pro-inflammatory effect upon glomerular meningeal cells, has been implicated in allograft rejection [25–27]. TGF beta is a key cytokine important in acute inflammation, it plays a central role in both the initiation and propagation of acute and chronic rejection in the acute rejection episodes of solid organ transplant recipients, TGF-β antibody has been reported to be capable in preventing allografts rejection [28–30]. Known to play an important role in activating TGF-β, Thrombospondin has long been considered a potent modulator of allografts rejection [31–33]. TIMP-1, a natural inhibitor of the MMPs, is also a key modulator in immune-rejection [34].

This study has several acknowledged limitations. As this is an observation study, future functional experiments are needed to investigate the underlying mechanism of specific cytokine related to the diseases. In addition, as this is an exploratory study, multiple testing in precaution of type I error should be performed in future study to validate the results. Furthermore, only a small fraction of cytokines was tested in this study, while it is known that there are hundreds or thousands molecules involved in the immunological responses of rejection and infection. Lastly, the current study evaluated the cytokine profiles statically for only one time-point, as the immunological responses of rejection and infection are complex and dynamically, the current findings should be further monitored and evaluated at different stages and time-points during development of diseases.

## Conclusions

This preliminary study demonstrated the disease-specific serological cytokine portraits in cardiac allograft rejection and pulmonary bacterial infection after heart transplant. A panel of serum cytokines that might have the potential to discriminate between cardiac rejection and lung infection was also identified. However the specific cytokine pathways and underlying mechanisms need further investigation.

## Abbreviations

ACUC: Animal Care and Use Committee
CINC-3: Cytokine-induced neutrophil chemoattractant 3
CNTF R Alpha: ciliary neurotrophic factor receptor alpha
FC: Fold change
FSL1: Follistatin-like 1
GFR alpha-1: glial cell line derived neurotrophic factor family receptor alpha 1
IFN-gamma: Interferon gamma
IL-13: Interleukin 13
IL-2: Interleukin 2
IL-2: Interleukin 2
IL-5: Interleukin 5
ISHLT: International Society of Heart and Lung Transplantation
IVC: inferior vena cava
MMP-2: Matrix metallopeptidase 2
NGFR: Nerve growth factor receptor
RAGE: Advanced glycosylation end-product specific receptor
TGF-beta1: Transforming growth factor beta 1
TGF-beta3: Transforming growth factor beta 3
TIMP-1: TIMP metallopeptidase inhibitor 1
